# “On-The-Spot” Arresting of Chondroitin Sulphate Proteoglycans: Implications for Ovarian Adenocarcinoma Recognition and Intervention

**DOI:** 10.3390/ijms17071136

**Published:** 2016-07-18

**Authors:** Priyamvada Pradeep, Yahya E. Choonara, Pradeep Kumar, Viness Pillay

**Affiliations:** Wits Advanced Drug Delivery Platform Research Unit, Department of Pharmacy and Pharmacology, School of Therapeutic Sciences, Faculty of Health Sciences, University of the Witwatersrand, Johannesburg, 7 York Road, Parktown 2193, South Africa; priyamvada.pradeep@wits.ac.za (P.P.); yahya.choonara@wits.ac.za (Y.E.C.); pradeep.kumar@wits.ac.za (P.K.)

**Keywords:** ovarian cancer, proteoglycans, crosslinked chondroitin sulphate, complexation, tumor proliferation, GD3G7 antibody, anti-VEGF

## Abstract

Ovarian Cancer (OC) is one of the leading causes of cancer-associated death among women. The underlying biochemical cause of OC proliferation is usually attributed to the over-expression of Chondroitin Sulphate Proteoglycans (CSPGs) wherein the CS-E subgroup plays a major role in tumor cell proliferation by over-expressing vascular endothelial growth factor (VEGF). We hereby hypothesize that by targeting the OC extracellular matrix using a CS-E-specific antibody, GD3G7, we could provide spatial delivery of crosslinkers and anti-VEGF agents to firstly induce in vivo crosslinking and complexation (arresting) of CS-E into a “biogel mass” for efficient and effective detection, detachment and reduction of tumorous tissue, and secondly inhibit angiogenesis in OC. It is further proposed that the antibody-assisted targeted delivery of CS-E crosslinkers can bind to highly anionic CS-E to form a polyelectrolyte complex to inhibit the formation of ovarian tumor spheroids that are responsible for spheroid-induced mesothelial clearance and progression of OC. The hypothesis also describes the potential in vivo “On-The-Spot” CSPG crosslinkers such as sodium trimetaphosphate (physical crosslinker), 1,12-diaminododecane (chemical crosslinker), poly(ethylene glycol) diglycidyl ether (synthetic polymer), and chitosan (natural polyelectrolyte-forming agent). In conclusion, this hypothesis proposes in vivo spatial crosslinking of CSPGs as a potential theranostic intervention strategy for OC—a first in the field of cancer research.

## 1. Introduction

Proteoglycans (PGs) are anionic macromolecules on the surface and extracellular matrix of cells that are responsible for structural assembly and regulation of various cellular processes [1]. Structurally, PGs comprise a protein core that is covalently bound to various unbranched polysaccharides collectively termed Glycosaminoglycans (GAGs), such as Chondroitin Sulphate (CS), Dermatan Sulphate (DS), Heparin Sulphate (HS), Keratin Sulphate (KS), and Hyaluronic Acid (HA) [1,2]. GAGs are linked to the protein core to form a molecular complex that plays a critical role in regulating cellular proliferation, apoptosis, migration, adhesion, invasion and extracellular matrix (ECM) assembly [3] via the highly anionic side chains of CS and DS, in particular. In addition, the up- or down-regulation and function of PGs along with enzymes involved in their biosynthesis have been associated with numerous types of pathological conditions including cancer [1,3].

**Figure 1:**
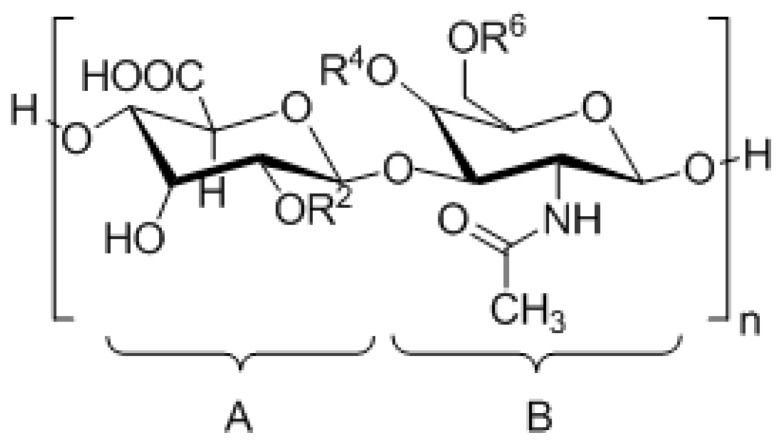
Disaccharide units of Chondroitin Sulphate (CS).

CS comprises alternating disaccharide units of d-glucuronic acid (auronic acid) and d-*N*-acetyl galactosamine (an amino sugar) (Figure 1) with a sulphate group present at C-2 of glucuronic acid and at C-4/C-6 of d-*N*-acetyl galactosamine. At instances, a few or all of the d-glucuronic acid residues are epimerized to l-iduronic acid to produce DS by the action of epimerase [2]. Based on the sulphation pattern, CS chains can be classified as either CS-A (GlcA-GalNAc(4-*O*-sulphate)), CS-B (or DS) (IdoA(2-*O*-sulphate)-GalNAc(4-*O*-Sulphate)), CS-C (GlcA-GalNAc(6-*O*-sulphate)), CS-D (GlcA(2-*O*-sulphate)–GalNAc(6-*O*-sulphate)) or CS-E (GlcA-GalNAc(4,6-*O*-disulphate)) (Table 1). These differential sulphation patterns enable specific interactions with various molecules within the ECM, on the cell membrane and intracellularly, such as with growth factors, cytokines, chemokines, adhesion molecules and lipoproteins [1,2].

**Figure 2:**
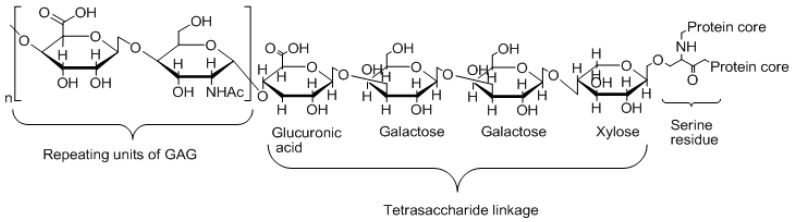
Typical proteoglycan structure depicting a core protein covalently bound to Glycosaminoglycan (GAG) chains via a tetrasaccharide linkage.

Furthermore, CS is attached to the core protein of the PGs via a serine residue using a tetrasaccharide linkage constituting xylose, two galactose molecules and GlcA (Figure 2). The biosynthesis of CS chains onto the proteinaceous core leads to the generation of Chondroitin Sulphate Proteoglycans (CSPGs) such as Aggrecan, Decorin and Veriscan (within the ECM), Syndecan and Glypicans (at the cell membrane) and Serglycin (intracellularly) which are essential for numerous biological functions such as cell proliferation, apoptosis, migration, adhesion and invasion, as well as ECM assembly, via their high negatively charged CS⁄DS side chains [3].

Structural modifications of CS in terms of the chain length, the sulphation pattern and the extent of epimerization may lead to an alteration of the biological properties [4]. The growth of tumor stroma is an initial characteristic of various solid tumors which is initiated by angiogenesis [4]. Angiogenesis is a process of formation of new blood vessels from the pre-existing vasculature and it is critical for providing oxygen and nutrients to the growing tumor [4]. Tumor stroma and fibrotic tissue have been reported to contain unusually higher concentrations of CSPGs compared to surrounding healthy tissues [3]. This has been noted in rectal, pancreatic, gastric, testicular, breast, ovarian and colon carcinomas, where CSPGs (in particular Versican and Decorin) were significantly increased. The sulphation pattern of CS also plays a crucial role in regulating several biological events with respect to growth factors and their receptors, enabling them to promote various biological events [5,6]. In healthy tissues, monosulphated disaccharide units are predominant, whereas in tumor tissues, non-, di- and tri-sulphated CS chains take over [7]. CS-E and CS-D, which are rare, and over-sulphated CS chains are found in pathological conditions such as cancer and their roles in growth factor–mediated signaling have been observed [8,9,10]. These CS chains are able to interact with various growth factors, such as heparin-binding growth factors, pleiotrophin (PTN), fibroblast growth factors (FGF), heparin-binding epidermal growth factor (BB-EGF), and vascular endothelial growth factor (VEGF), by binding with them through electrostatic interactions and sequesterization [9,10]. VEGF is the most significant growth factor when it comes to tumor-related angiogenesis [10]. Compared to non-neoplastic ECM, tumor-associated ECM contains higher quantities of over-sulphated GAGs and growth factors, especially VEGF [7].

## 2. Hypothesis

Variations in the sulphation pattern and types of GAGs have been reported, specifically in the case of Ovarian Cancer (OC) [7]. Contrary to normal ovaries, higher concentrations of CS-E (with increased VEGF concentrations) have been observed in OC and can be effectively detected and targeted by the tumor-specific antibody GD3G7 [11,12]. We hereby hypothesize that nano-archetypes conjugated with GD3G7 may be synthesized to deliver various crosslinkers, complexing agents and anti-VEGF agents to act in a two-way manner. Crosslinkers and complexing agents would specifically and locally crosslink (arrest) CS-E and/or form a polyelectrolyte complex. This would impede the proliferation of CS-E present in the tumor environment. In addition, the crosslinked “biogel mass” can then be easily detected, removed, or treated without affecting the surrounding healthy tissue. Simultaneously, anti-VEGF agents can control the action of VEGF and thus may further reduce angiogenesis. This nano-enabled system may be a promising strategy as a targeted theranostic system for OC intervention.

## 3. Evaluation of the Hypothesis

### 3.1. The Role of CS-E in Ovarian Cancer

**Figure 3:**
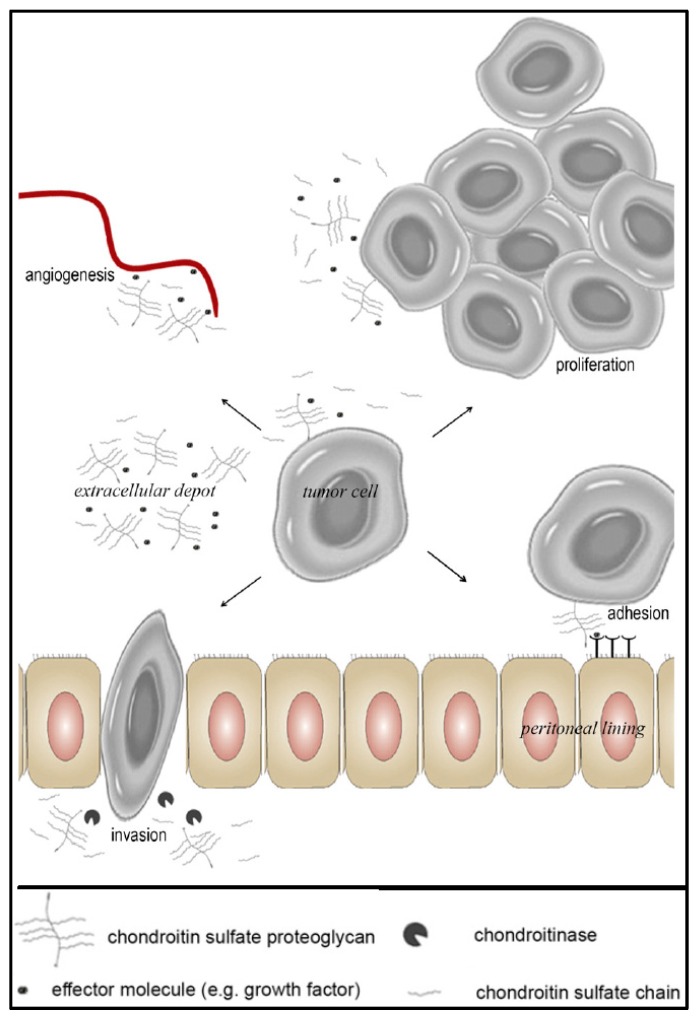
Processes in ovarian carcinogenesis in which chondroitin sulfate plays a critical role (Reference [7]; Reproduced with permission from Elsevier B.V. Ltd. © 2014).

CS-E has been shown to play a key regulating role in OC due to its angiogenesis function, tumor cell adhesiveness and migration [12]. The biosynthesis of CS-E is increased both in the tumor stroma as well as within neoplastic cells, resulting in profuse accumulation. The expression of mRNA for Gal-NAc-4-sulfate-6-*O*-sulfotransferase (GalNAc4S-6ST), an enzyme which is responsible for the biosynthesis of CS-E, is up-regulated in the case of OC [11]. CS-E is known to sequester very strongly with VEGF (an important angiogenic growth factor associated with tumor growth) through its 4-*O* and 6-*O* sulphate groups and hence leads to an increased VEGF concentration. An increase in the VEGF concentration further leads to neo-vasculatization in the tumor stroma and has been proven to be instrumental in ovarian spheroid formation (Figure 3) [11,12]. Furthermore, the formed tumor spheroid embeds into the mesothelial layer (a functional barrier for the spread of ovarian tumors) as well as on the walls of peritoneal and pleural cavity organs where integrin- and talin-dependent myosin and traction forces are used to encourage mesothelial cell displacement from beneath the spheroid and thus leads to further spread of cancer [13]. In addition, over-expression of CS-E leads to increased adhesiveness by adhesion molecules such as *N*-cadherin and *E*-cadherin which exacerbates tumor spheroid formation [12]. Therefore, CS-E-rich motifs can be employed as potential targets for OC therapy. Blockage, inhibition or enzymatic degradation of CS-E may inhibit tumor cell aggregation and metastasis, thus reducing the spread of OC [12].

### 3.2. CS-E Targeting with Antibody

**Figure 4:**
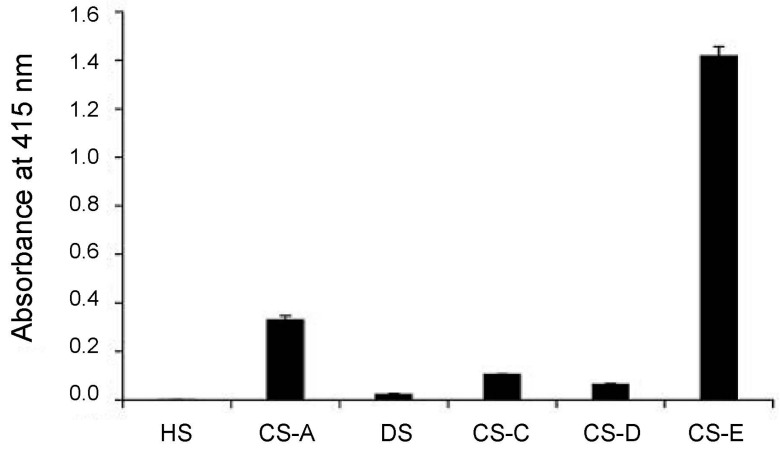
The reactivity of antibody GD3G7 with immobilized Heparin Sulphate (HS), CS-A, Dermatan Sulphate (DS), CS-C, CS-D, and CS-E was analyzed by indirect ELISA. Strong reactivity was observed with CS-E. Bars represent mean ± SD (*n* = 3). (Reference [11]; Reproduced with permission from Elsevier B.V. Ltd. © 2007).

GD3G7 is an antibody known to identify motifs abundant in CS-E epitopes which are strongly expressed in OC [11]. GD3G7 expression was primarily observed in the intra-tumoral stroma, the basal membrane zone underlying the tumor cells, and regions surrounding the blood vessels. Furthermore, a CS-E gradient was observed in regions where tumor tissue adjoins to non-tumorous regions (Figure 4) [11]. Hence, GD3G7 may be instrumental in identifying tumor-related CS-E. GD3G7 could also be employed in the therapy of OC by utilizing its “tumor-targeting” ability and hence could play a key role in targeted delivery of the hypothesized in vivo crosslinkers and anti-VEGF drugs such as bevacizumab.

### 3.3. Approaches to Target and Arrest CS-E Over-Expressing Tumorous Cells

The first approach is to counteract malignant tumor cells by forming an “On-The-Spot” Polyelectrolyte Complex (PEC) of anionic CS-E with a natural cationic polymer. CS-E is inherently anionic in nature and will form ionic bonds with a cationic polymer [14]. To this end, we propose the synthesis of nano-archetypes comprising chitosan (cationic polymer for PEC formation) conjugated with GD3G7 antibody (for targeting) and anti-VEGF agents (to prevent further spread of OC). This nanosystem would lead to the simultaneous inactivation of highly proliferative CS-E and the inhibition of VEGF at the tumorous site and could impede the progression of OC.

The second approach is to load the nano-archetypes with various biocompatible but non-biodegradable crosslinkers. Only a few compounds have been reported to crosslink with CS-GAGs to form a PEC. This phenomenon could be utilized in the targeted chemotherapy of OC. Nano-archetypes of compounds known to crosslink with CS-GAGs could be synthesized and then conjugated with GD3G7. Once at the tumor site (guided by the tagged antibody), the crosslinker would crosslink with CS-E to produce a hydrogel-based “biogel mass” that would become isolated from the tumorous vasculature and tumor growth could be terminated. In addition, due to its non-biodegradable nature and altered physicomechanical properties, the inherent in vivo rejection of the tumorous tissue may also be possible. Furthermore, the formation of such a complex hydrogel may interrupt the nutritional supply to the tumorous tissue, thereby preventing further tumor proliferation and growth.

**Scheme 1:**
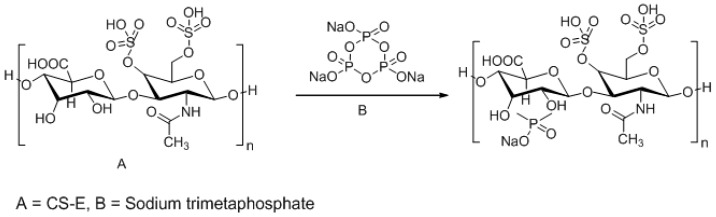
Proposed crosslinking reaction between CS-E and sodium trimetaphosphate (TMFS) [15].

A few crosslinkers have been identified to crosslink with CS. Sodium trimetaphosphate (TMFS) is a non-toxic compound used in the food industry to crosslink starch. The crosslinking reaction with TMFS involves binding of the hydroxyl group of CS as shown in Scheme 1 [15]. This leads to reduced affinity of CS towards water and renders it insoluble and inactive in vivo. This nanoparticle complex could then be targeted towards OC to halt the proliferation of CS-E and thus assist in preventing OC metastasis.

**Scheme 2:**
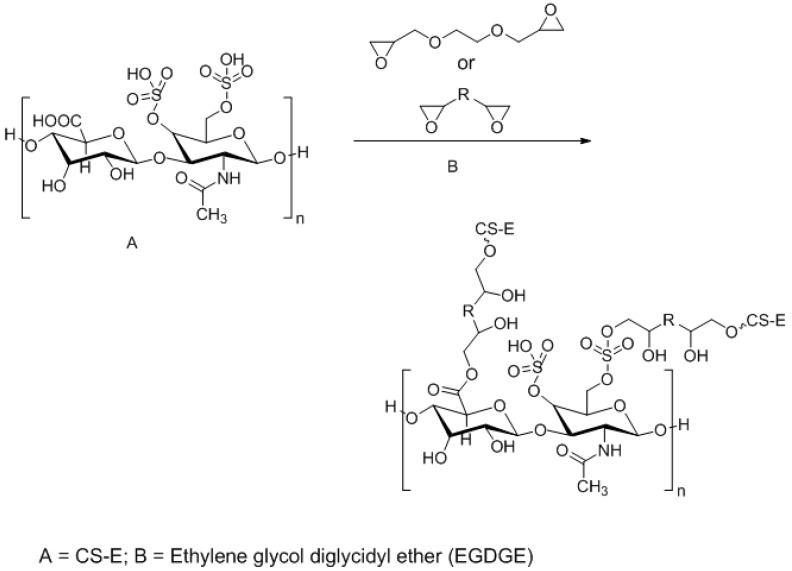
Proposed crosslinking reaction between CS-E and ethylene glycol diglycidyl ether (EGDGE) [16].

Ethylene glycol diglycidyl ether (EGDGE) has also been reported to crosslink with CS-E. The epoxy ring on EGDGE reacts with the carboxylate or ester sulphate group of CS, thus forming a hydrogel as depicted in Scheme 2 [16]. However, delivering a monomer in vivo could have safety implications. In line with the above findings, crosslinking of CS-E could be achieved using another epoxide-containing crosslinker, poly(ethylene glycol) diglycidyl ether (EX-810), which is known to directly crosslink with CS-E to form hydrogels with superior dimensional stability [17].

**Scheme 3:**
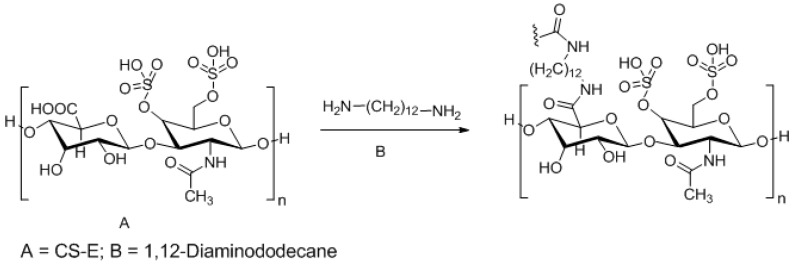
Proposed crosslinking reaction between CS-E and 1,12-diaminododecane [18].

Aliphatic compounds such as 1,12-diaminododecane have also been reported to crosslink with CS, forming a stable hydrogel with reduced water solubility and a lower degree of anionic groups. In this scenario, reaction occurs between the carboxylic acid of CS and the amine group of 1,12-diaminododecane to form a stable amide bond, thus forming a hydrogel of CS shown in Scheme 3 [18].

Based on the above discussion and differential reaction mechanisms of crosslinking vs. complexation, a third approach for the targeted delivery of crosslinkers at the site of OC could be employed through a bi-pronged approach involving “antibody-conjugated chitosan crosslinker” nano-achetypes. As per our hypothesis, this tripartite system would work in three dimensions at the cancerous site. Chitosan would form a PEC with the anionic charge of CS-E and the crosslinker will react with CS-E to form the inert hydrogel-based “biogel mass” to impede the function of CS-E and eventually obstruct the progression of OC.

## 4. Conclusions and Consequences of the Hypothesis

The hypothesized in vivo crosslinking and complexation of highly anionic CS side chains provides a novel concept for arresting the CS-E-mediated proliferation of tumorous ovarian tissue. The simultaneous incorporation of anti-VEGF agents into the nano-archetypes may further attenuate the VEGF already prevalent at the tumorous region, thereby providing an OC chemotherapeutic paradigm. In addition, with distinguishable physicomechanical properties, the crosslinked CS-E may potentially line the tumorous tissue and hence can be employed for the detection as well as removal of tumorous tissue. However, the targeted delivery of physical and chemical crosslinkers is an ardent task as the loading and release of such agents can be hindered by various formulation challenges. Although the crosslinkers proposed in this study are reported to be biocompatible, direct delivery and application of such crosslinkers in vivo should be considered only after detailed in vitro, in cyto, and ex vivo studies have been completed. We encourage (and urge) future researchers to apply feasible formulation techniques for the development of biocompatible crosslinker-payloads in the form of antibody-conjugated nano-archetypes capable of “accurately” targeting tumorous tissues in vivo.

## Figures and Tables

**Table 1:** Common sulphation patterns of Chondroitin Sulphate (CS) disaccharides. DS, Dermatan Sulphate.

CS Type	Disaccharide Unit	Substituents
CS-A	A = [β1-4]-d-glucuronic acid B = [β1-3]-*N*-acetyl-d-galactosamine	R^2^ = H R^4^ = 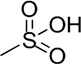 R^6^ = H
CS-B/DS	A = [β1-4]-l-iduronic acid B = [α1-3]-*N*-acetyl-d-galactosamine	R^2^ = H R^4^ = 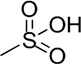 R^6^ = H
CS-C	A = [β1-4]-d-glucuronic acid B = [β1-3]-*N*-acetyl-d-galactosamine	R^2^ = H R^4^ = H R^6^ = 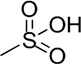
CS-D	A = [β1-4]-d-glucuronic acid B = [β1-3]-*N*-acetyl-d-galactosamine	R^2^ = 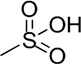 R^4^ = H R^6^ = 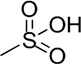
CS-E	A = [β1-4]-d-glucuronic acid B = [β1-3]-*N*-acetyl-d-galactosamine	R^2^ = H R^4^ = 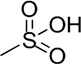 R^6^ = 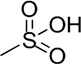
